# Studies on the Enhancement of Sparkling Wine and Chocolate Pairing through Compositional Profile Analysis

**DOI:** 10.3390/foods12183516

**Published:** 2023-09-21

**Authors:** Camelia Elena Luchian, Elena Cristina Scutarașu, Lucia Cintia Colibaba, Dragoș Grosaru, Valeriu V. Cotea

**Affiliations:** Faculty of Horticulture, Iași University of Life Sciences, 3rd M. Sadoveanu Alley, 700490 Iași, Romania; camelialuchian@uaiasi.ro (C.E.L.); cintia.colibaba@uaiasi.ro (L.C.C.); dgrosaru@gmail.com (D.G.); vcotea@uaiasi.ro (V.V.C.)

**Keywords:** sensory analysis, synergic match, chocolate, sparkling wines, food and beverage pairing

## Abstract

Consumers are looking to experience as many interesting culinary combinations as possible, and there is a growing tendency to associate wine with various foods. Although there are some studies associating wine with chocolate, especially red wine, no articles have been published referring to sparkling wines. Therefore, the purpose of this investigation was to identify the taste compatibility and sensory synergies between sparkling wine and chocolate, with a focus on identifying combinations that can enhance the tasting experience. For this experiment, 14 variants of sparkling wines obtained in Romania and 5 chocolate assortments were evaluated to identify the best culinary match. White chocolate fitted better with Chardonnay—demi-dry sparkling wine; ruby chocolate presented a good match with Fetească neagră—demi-dry; milk chocolate with 32% cocoa powder associated better with Tămâioasă românească—sweet; and dark chocolate with 70% and 95% cocoa powder had synergic matches with Fetească neagră—sweet. Wine attributes like sweetness, acidity, alcoholic strength and chocolate composition significantly impacted the level of match.

## 1. Introduction

Food and beverage pairing is an innovative and profitable strategy for the food and beverages sectors to meet consumer demands [1]. The existing literature provides some general aspects regarding the food and beverage pairing, but strategies to create the perfect match are still difficult [2]. 

Chocolate is a highly valued food product, obtained from cocoa solids, cocoa butter, sugar, lecithin and other ingredients [3]. The cocoa beans used to make chocolate are the fruits of cocoa trees (*Theobroma cacao L.*) that grow in Central and South America and in West Africa. Each bean is made of about 50% of a fat called cocoa butter. The beans have 20% protein and also contain water, as well as a large amount of nutrients, starch and other minerals [4]. The high content of compounds with nutritional value in cocoa, especially flavonoids with antioxidant and anti-inflammatory action, recommends chocolate as a food that should be part of a balanced diet [5]. Other cocoa constituents also increase the benefits of chocolate consumption, such as methylxanthines, namely theobromine and caffeine. Research in the field of pharmacology has identified abundant effects of these compounds that include central nervous system stimulation, diuresis, cardiovascular and metabolic effects, bronchial relaxation and increased secretion of gastric acids without producing addiction [6]. This product is consumed in high amounts all over the world, its taste being the main factor that influences consumers’ purchasing decisions. There are many different types of chocolate, and each has its own distinct taste and characteristics [7]. 

Sparkling wines have gained global popularity as the favorite beverage for special moments in life. The distinctive characteristics of these wines are closely related to the carbon dioxide content (approximately 600 kPa), which significantly influences the perception of consumers and the way of associating this drink with some foods. Most sparkling wines are obtained from colorless base wines with a relatively low alcohol content of up to 9% alcohol content and with a high acidity that enhances the sensory characteristics of the final product with an alcoholic concentration of approximately ≈12% alcohol content obtained after secondary fermentation. The production of red or rosé sparkling wines is relatively low due to current grape harvesting preferences. In order to be suitable for base wine, red grapes often do not reach the appropriate phenolic maturity that gives the wine sensory complexity [8]. Sparkling wines with high sugar content were usually associated with the consumption of desserts on festive occasions. However, there is a trend whereby current consumers prefer sparkling wines with very low sugar content before the meal. The acidity of these wines activates the gastric juices and their subtle flavors do not cover the sensory characteristics of the food [9].

The appearance of aroma constituents in sparkling wines, with a direct impact on their sensory characteristics, is affected by several factors, for example, the composition of the base wine, grape growing conditions, inoculated yeasts, stage of aging and winemaking practices. The sensory perception of sparkling wines is given by the interaction of different volatile constituents, while its character is influenced by effervescence, sweetness, acidity or bitterness and generated by non-volatile compounds that are soluble in water or a mixture of alcohols [10,11]. 

A food pairing can be considered successful when the mixture of two or more ingredients gives the taster a higher intensity of sensorial experience than any of those ingredients taken separately. Our sensorial inclinations are heavily influenced by childhood memories, when sensorial memory is constructed, traditional backgrounds, and daily customs, but also personal income [12]. However, there are some food combinations that always work, despite all these variables. 

The resemblances in cultivating conditions for both wine and chocolate make them ideal nominees for pairing. Wine and chocolate have many parallels in their production process, fermentation, aging, and blending. Both are complex and diverse, with a wide segment of aromas that can be experienced according to the variety. Wine has fruity notes, such as berries or citrus, as well as earthy tones like leather or tobacco. Chocolate also can boast notes of fruits but is more often defined as having nutty or spicy undertones. By understanding the production processes of each, one can further explore how they pair together to create a unique experience [12].

The association and correlation of flavors have attracted increased interest in recent times. One of the key approaches in this direction is based on the physical and chemical properties of food and the combinations of the existing chemical compounds. Some associations are the result of local history and culture. An alternative approach is based on the perception, relationship or interaction between the component stimuli (similarities, contrasts, harmonies, etc.). These approaches help consumers to better appreciate and understand food and beverage combinations and influence their purchasing decisions [13].

As with any wine pairing, the aim is to boost the characteristics of a particular wine and a particular food. Fine chocolate has a high cacao butter fat content, covering the palate and, in general, decreasing the print of any food that comes after. Certain wines have a relatively high acidity that can cut through the cocoa fat, setting up one’s mouth to fully enjoy the next bite of chocolate. In general, fruity wines are not able to accomplish this; the conflict between the astringency of the wine and the bitterness of the chocolate wrecks these pairings. Likewise, sweeter chocolates tend to be overwhelmed by sweet wines; high tannic dark chocolates are incompatible with high tannic wines, most of the times [14].

Several papers refer to wine and food pairings [15,16,17,18,19,20,21]. Wine is often associated with different types of meals (e.g., Chinese food) and alimentary products (e.g., cheese) [16]. Pimentel et al. [22] compared the flavonoid content of different assortments of chocolates and wines, but the focus was not on their sensory matching level. Most existent studies were using the hedonic method for the sensory approaches [15,23]. Donadini et al. [21] provided some recommendations for chocolate sample and beverage pairing (including balsamic vinegar, liqueur wine, coffee, and Port wine). No study was published on finding the best match between sparkling wines and chocolate. 

Therefore, the purpose of this paper was to identify the taste compatibility and sensory synergies between commercial sparkling wines (white and rosé) and chocolate samples, with a focus on identifying combinations that can enhance the tasting experience. For this aim, the specific objectives include the evaluation of the physicochemical characteristics of wines, individual analysis of the specific sensory properties of sparkling wines and chocolates, and evaluation of the degree of association between the different pairs of samples (chocolate–sparkling wine).

## 2. Materials and Methods

### 2.1. Samples Acquisition

For the experiment, 14 variants of commercial sparkling wines produced in the Dealurile Moldovei region (Romania) have been chosen. Of these, 10 assortments were obtained from white grape varieties (Chardonnay, Frâncușă, Grasă de Cotnari, Tămâioasă românească), while 4 of them were rosé sparkling wines produced from red varieties (Busuioacă de Bohotin, Fetească neagră). Analyzed wine samples were produced by SC Cotnari SA, Romania. Sparkling wines were characterized by different levels of reductive sugar (from brut nature to sweet). The chocolate samples were purchased from the commercial network, Lidl supermarket chain in Romania. All samples are produced by Rausch for J.D. Gross (Lidl). For this study, five assortments of chocolates were analyzed (white chocolate; ruby chocolate; milk chocolate, with 32% cocoa powder; dark chocolate, with 70% cocoa powder; dark chocolate, with 95% cocoa powder).

### 2.2. Physicochemical Characterisation of Sparkling Wine Samples

Physicochemical characterization of sparkling wine samples was conducted according to the International Organization of Vine and Wine (OIV) recommendations [24]. The following parameters were achieved: total acidity (g/L tartaric acid) and volatile acidity (g/L acetic acid) using titrimetric measurements; alcoholic strength (% vol.) by volume; pH and density (instrumental measurements); reductive sugar (g/L) using Luff-Schoorl assay.

### 2.3. Sensory Analysis

The sensory session was organized in accordance with the specifications indicated by the ISO8589:2010 [25] and ISO3591:1997 [26] standards and the OIV recommendations [27]. The sensory evaluation was carried out by a panel of 50 skilled tasters (28 men, 22 women, aged between 22 and 65) who had participated in previous wine-tasting sessions and were experienced in objectively evaluating samples. The next three steps were implemented over 3 consecutive days to diminish sensorial fatigue; also, 10 min breaks were taken every hour. All panel tasters were informed beforehand on the theme of the sensorial analysis and were aware of their ingestion of alcoholic beverages.

First of all, the chocolate samples were divided into small and equal pieces for each person and arranged on a small white plate. Chocolate samples were served at room temperature (20–21 °C). The tasters evaluated each type of chocolate (from white, ruby to dark) and completed a tasting sheet with some pre-established descriptors. Each attribute term was extensively described and explained to avoid any doubt about the relevant meaning. Sensory profiling of chocolate samples was realized according to Donadini et al. [21]. 

In the second part of the sensory evaluation, the sparkling wine samples were analyzed (in order of color, variety, and reductive sugar content). Sparkling wine samples were served at approximately 8 °C [27]. The intensity of each descriptor for both chocolate and sparkling wine was appreciated with notes from 0 (absent) to 5 (high intensity). Sensory profiling of the analyzed sparkling wines was made in accordance with common attributes that can be identified in variety and according to the producer’s specifications, such as olfactive notes (flowery, fruity, spices, etc.) and gustatory atributes (sweet, acid, bitter, etc.).

For the third part, sparkling wine samples were tasted in ascending order (from the lightest to the most complex wine) with each chocolate sample, and the perceived level of match was registered. The level of match was appreciated with notes from 0 (no match) to 10 (synergic match) for each pair of sparkling wine–chocolate samples. All the results were centralized and are presented in the paper as means plus standard deviation.

### 2.4. Statistical Analysis

Statistical analysis of the data was performed using XL-STAT Premium (Luminevo, Denver, CO, USA) for Principal Component Analysis (PCA) and STATGRAPHICS 19^®^ centurion (Statgraphics Technologies Inc., The Plains, VA, USA) for analysis of variance (ANOVA), post hoc analysis (Fisher LSD) and the coefficient of determination (R-Squared). For the PCA, 2 main components were extracted for the sparkling wine samples, represented by initial data variability and characterized by eigenvalues higher than or equal to 1.0. Each variable is represented in relation to its correlation with each of the components. The coefficient of determination (noted with R^2^) determines how well the data fit the regression model. Analysis of variance (ANOVA) uses the F-value to determine whether the between-group variability of means is larger than the within-group variability of the individual values. If the ratio of between-group and within-group variation is sufficiently large, it can be concluded that not all the means are equal. The Pr > F indicator (the *p*-value of the F statistic) shows how probable it is that the F value calculated from the test would have occurred if the null hypothesis of no difference among group means were true. Statistical tests were chosen in accordance with Donadini et al. [21]. 

## 3. Results and Discussion

The present study brings innovative elements: white and rosé sparkling wines are analyzed in parallel with white, ruby and dark chocolate according to their sensorial properties like aroma or mouthfeel. These indices, as far as the authors know, have not been analyzed until now. 

### 3.1. Physicochemical and Sensory Characterisation of Sparkling Wines

The physicochemical parameters (Table 1) of wines provide basic information in characterizing their quality. Table 2 and Table 3 illustrate the results obtained on the individual sensory characterization of sparkling wines. The analyzed samples were obtained from white (Chardonnay, Frâncușă, Grasă de Cotnari, Tămâioasă românească) and red varieties (Busuioacă de Bohotin, Fetească neagră), and were characterized by different reductive sugar levels (from 0.6 g/L of reductive sugar in P1—brut nature—to 48.2 g/L reductive sugar in P14—sweet) and alcoholic strengths from 10.4% vol. (P10) to 12.9% vol. (P3). The taste and appreciation of a wine depends on the overall balance of all components and individual consumer preferences [10]. Thus, residual sugar content has a significant influence on wines’ sensory balance. 

Samples with values above 12 g/L reductive sugar will usually be characterized as more balanced, while a higher content of sugar covers up the acidity and freshness [28]. This is confirmed by our results. For example, even if P8 samples presented the highest total acidity (Table 1), it was not perceived by the tasters due to its high level of sugar (31.6 g/L reductive sugar). According to Ailer et al. [28], the reductive sugar level accentuates the threshold concentrations of negative sensory descriptors like mousiness, atypical wine aging, oxidation or yeast decay. The possible negative attributes were not included in this study.

**Figure 1:**
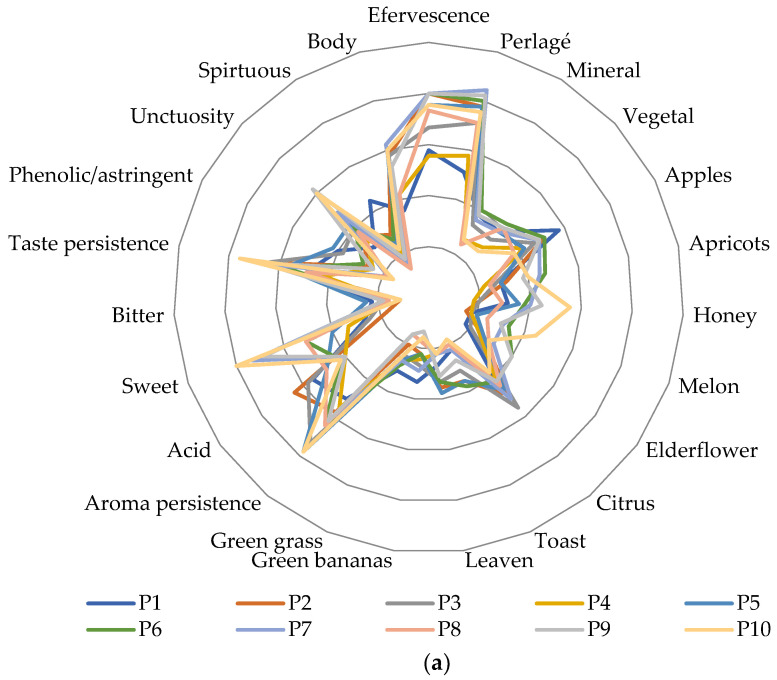

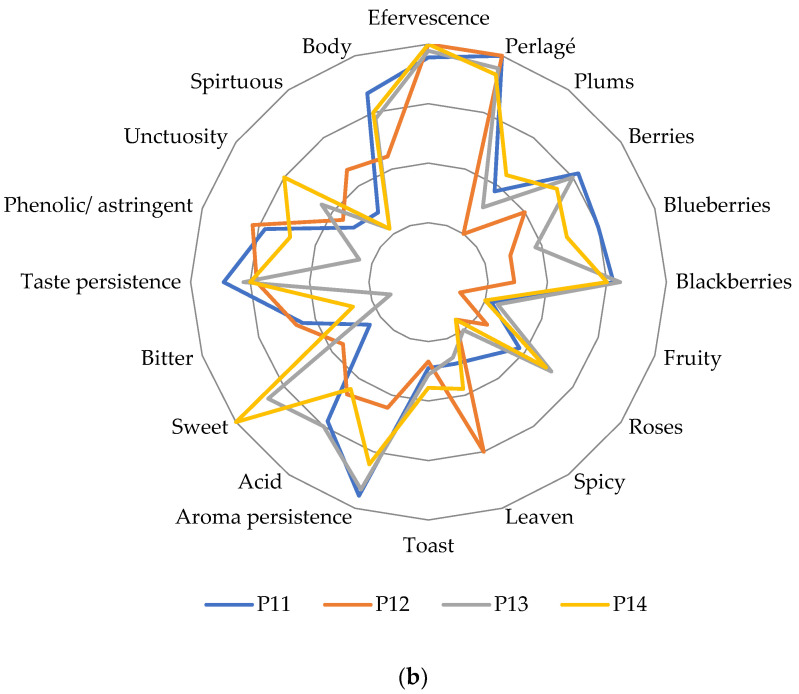
Sensory profiles of white (**a**) and rosé (**b**) sparkling wines. P1—Chardonnay, brut nature; P2—Frâncușă, brut;P3—Chardonnay, brut; P4—Grasă de Cotnari, brut; P5—Tămâioasă românească, brut; P6—Chardonnay, dry; P7—Chardonnay, demidry; P8—Grasă de Cotnari, demi dry; P9—Chardonnay, sweet; P10—Tămâioasă românească, sweet; P11—Fetească neagră, brut; P12—Busuioacă de Bohotin, dry; P13—Fetească neagră, demi dry; P14—Fetească neagră, sweet.

Ethanol levels can affect the sensory perception of wines by enhancing or masking some attributes [29]. According to King et al. [30], a wine’s sensory perception is also influenced by alcoholic strength. Thus, astringency perception is inversely proportional to ethanol and pH levels. Also, ethanol can influence sparkling wines’ bitterness. According to King et al. [30], samples in a lower alcoholic strength can be appreciated as having lighter fruity aromas, while high values intensify fruity attributes (ripe fruits). This can be confirmed by our results (Figure 1), but not in all cases. Therefore, for Chardonnay samples, P9 (11.0% vol.) presented lower notes for fruity aromas than P3 (12.9% vol.).

The total acidity of sparkling wine varied from 3.5 g/L tartaric acid in P9 and 4.8 g/L tartaric acid in P8. However, it is important to note that total acidity values can vary depending on the type of wine, grape variety used and specific production methods. 

An optimum amount of malic acid can contribute to a harmonious structure, influencing wine’s stability and improving its taste persistence. Malic acid can influence the final taste and perception of sparkling wines. Malic acid can add acidity, freshness and crispness to sparkling wines [10]. This is confirmed by our results in the case of P8 and P13, which were perceived as having a high acidity and freshness in relation to their highest malic acid contents (4 g/L and 3.9 g/L, respectively).

**Figure 2:**
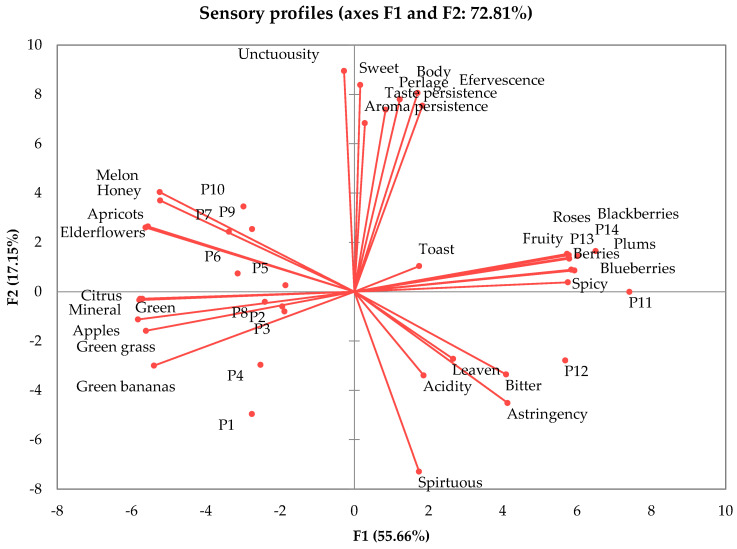
Principal component analysis on the sensory profile of sparkling wines. P1—Chardonnay, brut nature; P2—Frâncușă, brut;P3—Chardonnay, brut; P4—Grasă de Cotnari, brut; P5—Tămâioasă românească, brut; P6—Chardonnay, dry; P7—Chardonnay, demidry; P8—Grasă de Cotnari, demi dry; P9—Chardonnay, sweet; P10—Tămâioasă românească, sweet; P11—Fetească neagră, brut; P12—Busuioacă de Bohotin, dry; P13—Fetească neagră, demi dry; P14—Fetească neagră, sweet.

Following the PCA analysis (Figure 2), two principal components were extracted for sparkling wine samples, which summarized 72.81% of the data variability. From Figure 2, can be observed that each variable (sensory descriptors) is represented in relation to its correlation with each of the components.

The samples are grouped in different quadrants in Figure 2, depending on the predominant sensory descriptors. Therefore, the P1-P4 and P8 samples are more vegetal, with light green banana and apple aromas, while in P6, P7, P9 and P10, ripe fruits and sweet notes were predominant (apricots, honey, melon). The higher acidity was perceived by the tasters in the P2 and P13 variants, while the sweet taste was more intense in P10 and P14. A strong astringency was identified in the P12 variant, while the lowest notes were given for the P10 sample. Regarding rosé sparkling wines, P11 was characterized by its intense aroma of berries, plums and intense spicy notes, P11 by its intense leaven odor and spirituous taste sensation, P13 presented strong blackberry notes, while P14 had intense notes of plums and roses. Of the analyzed sparkling wine samples, P2 had the highest perceived acidity, P12 and P11 showed the more intense bitter taste, while P10 and P14 were perceived as the sweetest. Regarding their effervescence, P2 and P6 received the greatest notes, while P4 the fewest. From PCA analysis, some correlation between sensory attributes can be observed. So, the notes received for citrus, green apples, green banana and green grass attributes were proportional. Aroma and taste persistence are dependent on the sample’s sweetness and body. Thus, P10 samples had the longest aroma and taste persistence, being an aromatic variety and due to its high level of reductive sugar. Sparkling wines’ acidity is proportional to bitterness and astringency.

A significant difference between the samples was obtained for the following descriptors: sweet taste, aroma persistence, taste persistence, astringency and unctuosity (*p* < 0.05) for white sparkling samples (Table 2). Rosé variants were significantly differentiated by blackberry notes, a leaven aroma, aroma persistence, bitter and sweet tastes, and astringency (Table 3). The homogenous groups are indicated by superscript letters.

### 3.2. Characterisation of Chocolate Samples

**Figure 3:**
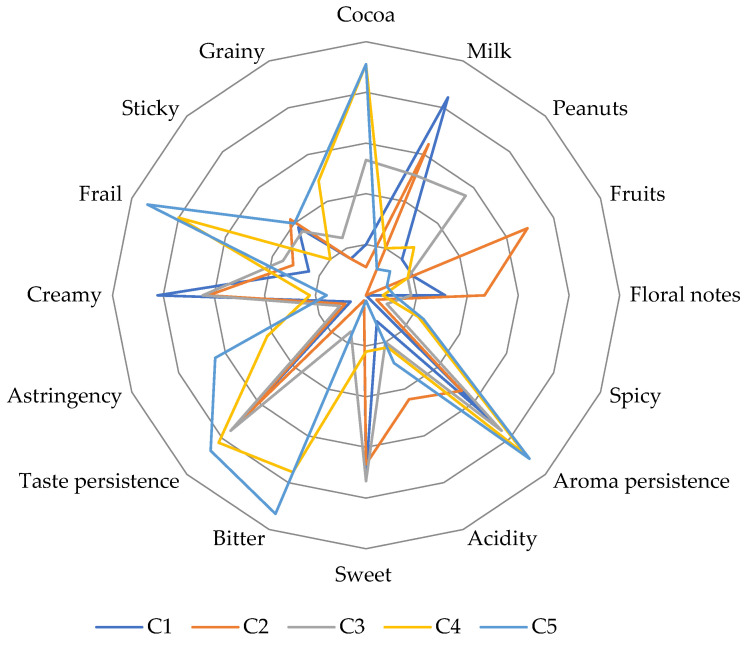
Sensory profiles of chocolate samples. C1—white chocolate; C2—ruby chocolate; C3—milk chocolate, with 32% cocoa powder; C4—dark chocolate, with 70% cocoa powder; C5—dark chocolate, with 95% cocoa powder.

For this experiment, five assortments of chocolate (white, ruby, milk and dark—with 32%, 70%, 95% cocoa), with variable fat (from 36.1 g/100 g in C1 to 51.1 g/100 g in C5) and carbohydrate content (from 12.7 g/100 g in C5 to 53.1 g/100 g in C2) were analyzed. The ingredients and nutritional information provided by producers are presented in Table 4 (not determined in this study). According to sensory analysis results (Figure 3), C1 was appreciated as the creamiest, with an intense aroma of milk and a distinct sweetness. Floral notes were better perceived in the C2 variant, which is also characterized as sticky and acidic. In relation to its high level of sugar, the C3 sample was perceived as sweetest, but with intense fruit and peanuts aromas. Due to their high concentration of cocoa powder, C4 and C5 received the highest notes for bitter, astringent, frail and grainy descriptors.

In accordance with the results of Thamke et al. [31], samples with a higher proportion of cocoa powder were perceived as the most bitter and astringent, while samples with low content or without cocoa were described by sensory descriptors belonging to the sweet cluster.

**Figure 4:**
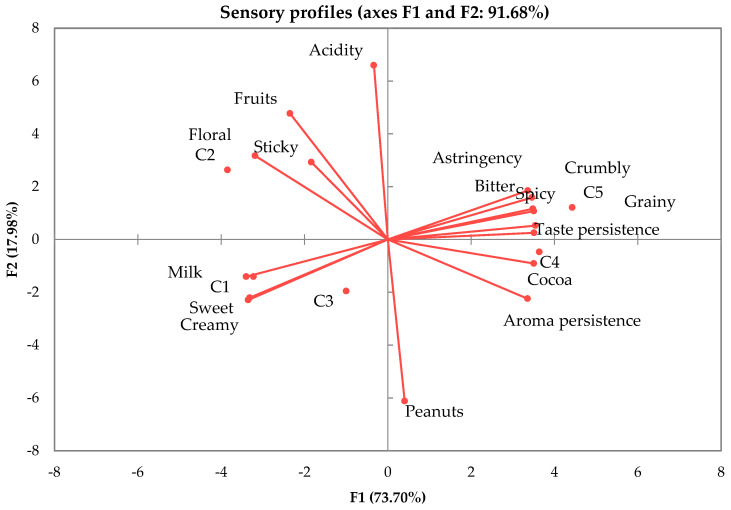
Principal component analysis on the sensory profile of sparkling wines. C1—white chocolate; C2—ruby chocolate; C3—milk chocolate, with 32% cocoa powder; C4—dark chocolate, with 70% cocoa powder; C5—dark chocolate, with 95% cocoa powder.

From Table 3, a significantly differentiation (*p* < 0.05) can be observed between the analyzed samples regarding the most sensory attributes (cocoa, milk, peanuts, fruits, floral, aroma persistence, sweet and bitter taste, astringency, crumbly). For PCA analysis (Figure 4), two components (F1 and F2) were extracted, totaling 91.68% of data variability. The samples are grouped in different quadrants, depending on the predominant sensory descriptors. It can be observed that milk aroma is proportional to the creamy attribute, acidity is dependent on fruity notes, and bitter taste contributes to higher taste persistence. 

The notes accorded to aroma persistence are generally correlated with the cocoa powder proportions used. Aroma and taste persistence are related to cocoa proportion. Similar results were presented by Donadini et al. [21], who postulated that the creamy character is perceived as higher as the cocoa powder level decreases. Moreover, consumers’ taste receptors are more sensitive to bitter tastes in comparison to sweet tastes [32].

### 3.3. Association Degree between Sparkling Wine and Chocolate

**Figure 5:**
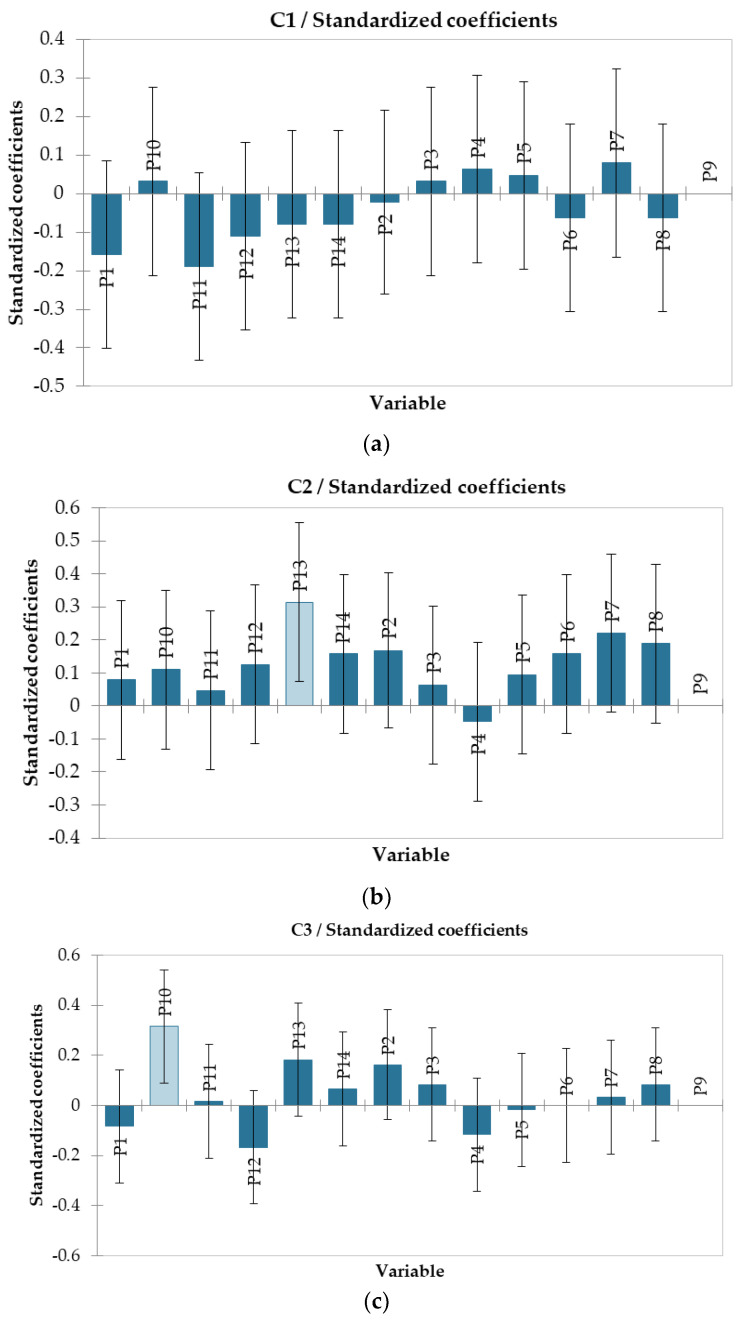

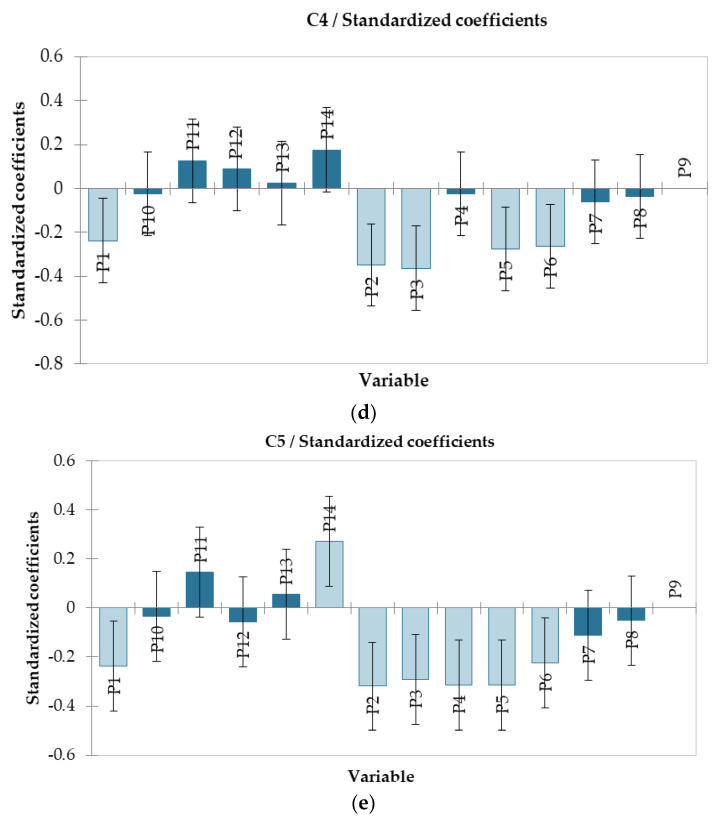
Standardized coefficients on association of chocolate samples with sparkling wine samples. C1—white chocolate (**a**); C2—ruby chocolate (**b**); C3—milk chocolate (**c**), with 32% cocoa powder; C4—dark chocolate, with 70% cocoa powder (**d**); C5—dark chocolate, with 95% cocoa powder (**e**); P1—Chardonnay, brut nature; P2—Frâncușă, brut; P3—Chardonnay, brut; P4—Grasă de Cotnari, brut; P5—Tămâioasă românească, brut; P6—Chardonnay, dry; P7—Chardonnay, demidry; P8—Grasă de Cotnari, demi dry; P9—Chardonnay, sweet; P10—Tămâioasă românească, sweet; P11—Fetească neagră, brut; P12—Busuioacă de Bohotin, dry; P13—Fetească neagră, demi dry; P14—Fetească neagră, sweet.

This research searches the predictability and potential associations between sparkling wines and chocolates using a sensory approach. Both sparkling wine and chocolate are appreciated by consumers for their distinct and complex sensory characteristics, and their combination can provide a special culinary experience. In Figure 5, the correlation and perception of each chocolate product varies according to the wine it was paired with. Data in Table 5 reach the same output, focusing on the principle that wine’s sensorial profile influences consumer’s perception and preference in quantitative evaluation of the pairings. Pittigrew and Charters [33] are of the opinion that wine is strongly paired with food with complementary attributes. By applying standardized sensory evaluation methods and techniques, the sensory characteristics of sparkling wine and chocolate, such as sweetness, acidity, flavors and texture, were evaluated. The results of this research contribute to a better understanding of taste interactions between sparkling wine and chocolate. It is important to note that preferences and taste perceptions may vary from one individual to another and the results can only provide general indications and guidelines regarding associations. In Table 5, homogenous groups can be observed according to post hoc comparison at a significance level of *p* ≤ 0.05. Thus, for C3, C4, and C5 samples, significant differences were registered regarding their association with sparkling wines. 

According to Table 5, the high degree of sweetness, unctuousness and creaminess from the white chocolate (C1) fitted better with P7 sparkling wine (7.22 ± 0.83) (Chardonnay, demi-dry), which reveals a complex and fruity profile, with accents of honey, elderberry and melon. These sensory characteristics balance harmoniously with the sweet taste of chocolate, resulting in an exceptional taste synergy. White chocolate is not considered suitable in association with P11 sparkling wine (Fetească neagră—brut), which is characterized by persistent berries notes.

Ruby chocolate (C2) was mentioned for its strong fruity nuances. It offers a distinct sensory experience due to its slightly acidic taste and unctuous texture. Its combination with P13 wine (8.22 ± 1.30) (Fetească neagră—demi-dry) has the highest note, thus ensuring a gustatory harmony, attributed to their fruity and floral characters (blackberries, red fruits, plums, roses, etc.). On the other hand, this chocolate flavor was not appreciated in association with sample P4 (Grasă de Cotnari—brut). 

The C3 variant (milk chocolate, with 32% cocoa powder) was appreciated for its creamy texture and sweet taste. This assortment was paired with P10 (8.73 ± 1.12) (Tămâioasă românească—sweet), as it shares similar sensory characteristics. This sparkling wine sample stands out for its distinct aromas of melon and honey, which balance perfectly with the sweetness and creaminess of the chocolate. From sensory analysis, it can be concluded that the intense astringency and pronounced bitterness of P12 (Busuioacă de Bohotin—dry) matched the least with C3. In general, when associating food and drink, harmony and balance between aromas, tastes and characteristics are sought [34].

C4 chocolate (dark chocolate, with 70% cocoa powder) had an excellent match with most sparkling wines. The high concentration of cocoa gives a rich aroma profile and great persistence. This assortment presented a synergistic match with P14 (8.33 ± 0.71) (Fetească neagră—sweet). This sparkling wine was remarked for its fruity aromas (blackberries and plums), which perfectly complement the intense cocoa notes of the chocolate. The minerality, vegetal character and intense citrus aromas of P2 (Frâncușă—brut) did not fit with the sensory properties of C4 chocolate. Thus, differences in flavors and taste profiles can interfere and not match in a pleasant way.

The C5 sample (dark chocolate, with 95% cocoa powder) was characterized by high astringency, bitterness and pronounced graininess. These features make this chocolate a suitable choice for those who appreciate an intense and cocoa-rich taste experience. Regarding its degree of association with sparkling wines, the highest score was given to P14 sample (8.11 ± 0.93) (Fetească neagră—sweet). The complex flavors of blackberries and plums counterbalance the astringency of the chocolate and contribute to its balance. The combination of the sweet note and the fruity aromas of the sparkling wine with the high intensity of the bitter taste of chocolate ensure a pleasant and satisfying gustatory harmony. On the other hand, the other types of sparkling wine hardly combine at all because of its intense and astringent notes. The specific characteristics of this chocolate can accentuate certain undesirable aspects in combination with other sparkling wines, leading to a taste imbalance and a less pleasant experience. Tastes and taste preferences may vary from one taster to another [34]. Ideal combinations can be subjective and depend on one’s personal preferences. The recommendation to combine sample C5 with P14 (Fetească neagră—sweet) is based on their complementary characteristics and flavors, but it is always useful to experiment and discover combinations that suit individual tastes. According to Bastian et al. [17], consumers appreciate more the pairs of samples in which the unpleasant flavors are suppressed. Keast and Breslin [35] showed that bitterness and sweetness are suppressive. 

The coefficient of determination (Table 6) shows the proportion in which the sparkling wine’s perception is affected by chocolate’s sensory characteristics. Low values of this parameter explain the variability of the data. For example, in the case of C5, the R^2^ value indicates that 49% of the variance of the chocolate’s sensory descriptors is explained by the sparkling wine’s properties. 

Table 7 shows that the received notes for the association of C1 and C2 with sparkling wines were similar between samples, while for C3, C4 and C5 the null hypothesis can be rejected. By applying Wilks’ Lambda test, the association between sparkling wines and chocolate is dependent on each product’s sensory characteristics.

According to the results, demi-dry sparkling wines matched better with most of the analyzed chocolate assortments, probably due to their level of total sugar. The wine sample that paired the best was P14. Harrington et al. [20] postulated that acidity, sweetness and tannins of wines, but also the cheese fattiness, significantly affected the level of match between wine and cheese samples. Rosé sparkling wines were more versatile for pairing with chocolate. C5 chocolate had the lowest notes in association with most of the sparkling wines. This can be due to the fact that the sweetness of chocolate increased the perception of bitterness and astringency of the sparkling wine sample [34]. Both wine and chocolate are bonded with emotional concepts generated by sensory characteristics and can have a positive impact on consumers [23]. On the other hand, for many consumers, taste depends on experience, customs, culture, and personal preferences [36]. For most of the sparkling wine samples, pairings with C5 chocolate were seldom appreciated, and were less associated than pairings with the other chocolates. The results are in accordance with those postulated by Donadini et al. [21]. The authors suggested that the generalized dislike of pairings with the chocolates with high proportions of cocoa may depend on the extreme bitterness and astringency, that, historically and genetically, evoke a classic rejection response [37]. Consumers generally dislike foods with extreme sensory attributes [38]. This study provides essential data to implement a strategic approach that links consumer behavior.

## 4. Conclusions

Limited data are available on the ideal match between sparkling wine and chocolate. A successful sparkling wine–chocolate pairing is based on meeting the necessities of consumers and creating a pleasant experience beyond their expectations. This study can act as a possible guide for the general consumer in understanding the principles behind wine pairing, in this case, sparkling wine with chocolate. Sparkling wines with fine *perlagé* and balanced acidity can counterbalance the sweetness of chocolate and amplify its rich and complex taste. This pairing creates an interesting synergy between the fruity and floral notes of the wine and the aromatic and creamy profile of the chocolate, leading to a delicious taste experience. According to the results, white chocolate fitted better with Chardonnay—demi-dry sparkling; ruby chocolate presented a good match with Fetească neagră—demi-dry; milk chocolate with 32% cocoa powder fitted better with Tămâioasă românească—sweet; while dark chocolate with 70% and 95% cocoa powder had synergistic matches with Fetească neagră—sweet. Wine attributes like sweetness, acidity, alcoholic strength and chocolate composition significantly impacted the level of match.

## Figures and Tables

**Table 1:** Physicochemical parameters of sparkling wines.

Code	Sparkling Wine Samples	Reductive Sugars g/L	Alcoholic Strength % vol.	Total Acidity g/L Tartaric Acid	Malic Acid g/L	Lactic Acid g/L	Volatile Acidity g/L Acetic Acid	Density	pH
P1	Chardonnay–Brut nature	0.6 ± 0.02	12.3 ± 0.00	3.6 ± 0.00	1.0 ± 0.01	0.9 ± 0.01	0.28 ± 0.01	0.9898 ± 0.01	3.23 ± 0.02
P2	Frâncușă–Brut	9.3 ± 0.01	11.4 ± 0.01	4.1 ± 0.00	2.0 ± 0.00	0.0 ± 0.00	0.26 ± 0.01	0.9941 ± 0.02	3.01 ± 0.01
P3	Chardonnay–Brut	5.8 ± 0.03	12.9 ± 0.00	3.8 ± 0.00	1.1 ± 0.00	1.1 ± 0.01	0.26 ± 0.00	0.9919 ± 0.00	3.22 ± 0.01
P4	Grasă de Cotnari–Brut	10.6 ± 0.01	12.1 ± 0.01	4.5 ± 0.01	3.5 ± 0.02	0.0 ± 0.00	0.31 ± 0.00	0.9952 ± 0.01	3.27 ± 0.00
P5	Tămâioasă românească–Brut	8.8 ± 0.00	10.9 ± 0.00	4.0 ± 0.01	2.0 ± 0.02	0.1 ± 0.00	0.37 ± 0.01	0.9944 ± 0.00	3.21 ± 0.01
P6	Chardonnay–Dry	19.7 ± 0.00	11.2 ± 0.00	3.6 ± 0.00	1.1 ± 0.00	0.9 ± 0.01	0.24 ± 0.01	0.9987 ± 0.00	3.26 ± 0.00
P7	Chardonnay–Demidry	32.2 ± 0.02	11.8 ± 0.00	3.8 ± 0.00	1.3 ± 0.00	0.7 ± 0.00	0.25 ± 0.01	1.0034 ± 0.00	3.30 ± 0.01
P8	Grasă de Cotnari–Demi dry	31.6 ± 0.02	11.7 ± 0.00	4.8 ± 0.00	4.0 ± 0.00	0.0 ± 0.00	0.33 ± 0.01	1.0047 ± 0.01	3.30 ± 0.01
P9	Chardonnay–Sweet	51.4 ± 0.00	11.0 ± 0.01	3.5 ± 0.00	1.2 ± 0.01	0.5 ± 0.00	0.20 ± 0.01	1.0116 ± 0.00	3.37 ± 0.00
P10	Tămâioasă românească- Sweet	46.9 ± 0.01	10.4 ± 0.01	3.7 ± 0.00	1.2 ± 0.00	0.2 ± 0.01	0.28 ± 0.00	1.0107 ± 0.00	3.17 ± 0.01
P11	Fetească neagră–Brut	9.0 ± 0.01	11.0 ± 0.00	4.0 ± 0.01	2.8 ± 0.00	0.0 ± 0.00	0.3 ± 0.00	0.9944 ± 0.00	3.17 ± 0.00
P12	Busuioacă de Bohotin–Dry	15.0 ± 0.00	11.6 ± 0.01	4.2 ± 0.01	2.4 ± 0.01	0.0 ± 0.00	0.28 ± 0.01	0.9963 ± 0.00	3.10 ± 0.01
P13	Fetească neagră–Demi dry	30.3 ± 0.02	11.6 ± 0.00	4.3 ± 0.00	3.9 ± 0.01	0.0 ± 0.00	0.37 ± 0.01	1.0041 ± 0.01	3.39 ± 0.01
P14	Fetească neagră–Sweet	48.2 ± 0.02	11.3 ± 0.01	4.2 ± 0.01	2.3 ± 0.00	0.0 ± 0.00	0.29 ± 0.01	1.0108 ± 0.01	3.12 ± 0.00

The physicochemical parameters were evaluated in triplicates and the results are expressed by mean and standard deviation.

**Table 2:** Sensory characteristics of white sparkling wines.

Sensory Descriptors	P1	P2	P3	P4	P5	P6	P7	P8	P9	P10	*p*-Value
Efervescence	2.89 ± 1.54 ^a^	4.00 ± 0.87 ^b^	3.33 ± 1.32 ^ab^	2.78 ± 1.48 ^a^	3.78 ± 1.30 ^ab^	4.00 ± 0.71 ^b^	4.00 ± 0.71 ^b^	3.67 ± 0.87 ^ab^	4.00 ± 0.71 ^b^	3.78 ± 1.09 ^ab^	0.1283
Perlage	2.56 ± 1.59 ^a^	3.89 ± 1.05 ^bc^	3.56 ± 1.42 ^abc^	2.89 ± 1.76 ^ab^	3.89 ± 1.36 ^bc^	4.00 ± 1.50 ^bc^	4.22 ± 0.83 ^c^	3.56 ± 1.24 ^abc^	4.11 ± 0.93 ^bc^	3.78 ± 1.30 ^abc^	0.1755
Mineral	1.89 ± 0.78 ^a^	2.00 ± 1.00 ^a^	1.67 ± 0.71 ^a^	1.33 ± 0.87 ^a^	1.78 ± 0.83 ^a^	2.00 ± 1.80 ^a^	1.78 ± 1.20 ^a^	1.22 ± 0.83 ^a^	1.89 ± 1.54 ^a^	1.33 ± 1.41 ^a^	0.8165
Green	1.89 ± 1.27 ^a^	2.11 ± 1.27 ^a^	1.67 ± 1.00 ^a^	1.44 ± 0.88 ^a^	1.89 ± 1.05 ^a^	2.11 ± 1.69 ^a^	1.89 ± 0.93 ^a^	2.00 ± 1.00 ^a^	2.00 ± 1.12 ^a^	1.33 ± 1.12 ^a^	0.8799
Apples	2.88 ± 1.13 ^a^	2.56 ± 1.33 ^a^	2.33 ± 1.00 ^a^	2.11 ± 0.93 ^a^	2.11 ± 1.17 ^a^	2.56 ± 1.13 ^a^	2.44 ± 0.73 ^a^	1.89 ± 1.05 ^a^	2.44 ± 0.73 ^a^	1.89 ± 1.36 ^a^	0.7032
Apricots	1.44 ± 1.42 ^a^	1.56 ± 1.59 ^a^	1.33 ± 1.50 ^a^	1.11 ± 1.05 ^a^	1.44 ± 1.59 ^a^	2.33 ± 1.80 ^a^	2.22 ± 0.97 ^a^	1.22 ± 0.97 ^a^	1.89 ± 1.05 ^a^	2.00 ± 1.32 ^a^	0.5384
Honey	1.56 ± 1.51 ^abc^	1.00 ± 1.12 ^ab^	1.00 ± 0.87 ^ab^	0.89 ± 0.78 ^a^	1.78 ± 1.72 ^abc^	1.89 ± 1.45 ^abc^	2.00 ± 1.22 ^abc^	1.44 ± 1.01 ^ab^	2.22 ± 1.64 ^bc^	2.78 ± 1.56 ^c^	0.0707
Melon	0.89 ± 1.62 ^a^	0.78 ± 1.30 ^a^	0.89 ± 0.78 ^a^	0.89 ± 0.78 ^a^	1.00 ± 1.00 ^a^	1.67 ± 1.22 ^ab^	1.56 ± 1.51 ^ab^	1.22 ± 0.67 ^ab^	1.50 ± 1.31 ^ab^	2.22 ± 1.86 ^b^	0.2929
Elderflowers	0.89 ± 1.05 ^a^	1.33 ± 1.22 ^ab^	1.00 ± 1.12 ^ab^	1.22 ± 1.09 ^ab^	1.33 ± 1.00 ^ab^	2.00 ± 1.32 ^b^	1.56 ± 1.13 ^ab^	1.44 ± 1.01 ^ab^	2.00 ± 1.12 ^b^	1.44 ± 1.13 ^ab^	0.4846
Citrus	2.11 ± 1.27 ^a^	2.44 ± 1.33 ^a^	2.78 ± 0.67 ^a^	2.11 ± 1.17 ^a^	2.33 ± 1.32 ^a^	2.11 ± 1.17 ^a^	2.56 ± 1.01 ^a^	2.22 ± 0.97 ^a^	2.11 ± 0.60 ^a^	1.89 ± 1.45 ^a^	0.8786
Toast	1.11 ± 0.78 ^a^	1.78 ± 1.39 ^a^	1.56 ± 1.51 ^a^	1.11 ± 0.78 ^a^	1.78 ± 1.20 ^a^	1.89 ± 1.17 ^a^	1.11 ± 0.78 ^a^	1.00 ± 1.12 ^a^	1.33 ± 1.58 ^a^	0.89 ± 0.78 ^a^	0.5198
Leaven	1.33 ± 0.87 ^a^	1.78 ± 1.48 ^a^	1.67 ± 1.12 ^a^	1.11 ± 1.05 ^a^	1.89 ± 1.05 ^a^	1.67 ± 1.41 ^a^	1.11 ± 0.93 ^a^	1.11 ± 1.05 ^a^	1.56 ± 1.33 ^a^	1.11 ± 0.93 ^a^	0.7319
Green bananas	1.67 ± 1.58 ^a^	1.11 ± 1.17 ^a^	1.33 ± 1.12 ^a^	1.22 ± 1.09 ^a^	1.11 ± 1.17 ^a^	1.11 ± 0.93 ^a^	1.44 ± 1.13 ^a^	0.89 ± 0.60 ^a^	0.67 ± 0.71 ^a^	0.78 ± 1.09 ^a^	0.7038
Green grass	1.56 ± 1.51 ^b^	1.00 ± 1.00 ^ab^	1.22 ± 0.67 ^ab^	1.33 ± 0.87 ^ab^	1.33 ± 0.87 ^ab^	1.44 ± 1.59 ^ab^	1.33 ± 0.71 ^ab^	0.78 ± 0.83 ^a^	0.78 ± 0.83 ^a^	1.22 ± 1.39 ^ab^	0.6656
Aroma persistence	2.56 ± 1.33 ^a^	2.89 ± 0.93 ^abc^	3.67 ± 0.71 ^bc^	2.78 ± 0.83 ^ab^	3.89 ± 1.05 ^c^	3.22 ± 1.48 ^abc^	3.11 ± 1.45 ^abc^	3.22 ± 0.83 ^abc^	3.11 ± 0.93 ^abc^	3.89 ± 0.60 ^c^	0.0451 *
Acidity	2.78 ± 0.83 ^ab^	3.22 ± 1.09 ^b^	2.89 ± 0.78 ^ab^	2.00 ± 1.12 ^a^	2.44 ± 0.73 ^ab^	2.11 ± 0.60 ^a^	2.00 ± 1.00 ^a^	2.44 ± 1.01 ^ab^	2.00 ± 1.00 ^a^	2.11 ± 1.36 ^a^	0.0823
Sweet	1.33 ± 1.22 ^ab^	0.89 ± 0.78 ^a^	1.22 ± 1.39 ^a^	1.67 ± 1.66 ^ab^	2.00 ± 1.22 ^ab^	2.56 ± 1.33 ^bc^	3.67 ± 0.87 ^cd^	2.56 ± 1.33 ^bc^	3.44 ± 1.51 ^cd^	4.00 ± 1.66 ^d^	0.0000 *
Bitter	1.11 ± 0.33 ^a^	0.56 ± 0.73 ^a^	0.89 ± 1.27 ^a^	0.78 ± 1.09 ^a^	1.22 ± 0.97 ^a^	0.78 ± 0.97 ^a^	0.89 ± 0.78 ^a^	0.78 ± 1.09 ^a^	0.89 ± 1.27 ^a^	0.56 ± 0.73 ^a^	0.9145
Taste persistence	2.44 ± 1.01 ^ab^	3.44 ± 0.73 ^c^	3.33 ± 1.00 ^bc^	2.11 ± 1.17 ^a^	2.89 ± 1.05 ^abc^	2.89 ± 0.93 ^abc^	3.22 ± 1.20 ^bc^	2.44 ± 1.24 ^ab^	3.33 ± 1.12 ^bc^	3.78 ± 0.83 ^c^	0.0226 *
Astringency	2.00 ± 1.00 ^bc^	1.33 ± 0.87 ^abc^	1.89 ± 0.78 ^bc^	1.22 ± 0.67 ^ab^	2.11 ± 0.93 ^c^	1.44 ± 1.24 ^abc^	0.89 ± 0.78 ^a^	0.89 ± 1.05 ^a^	1.22 ± 0.97 ^ab^	0.78 ± 0.97 ^a^	0.0202 *
Unctuosity	1.44 ± 0.53 ^a^	1.78 ± 0.83 ^ab^	2.00 ± 1.12 ^ab^	1.67 ± 0.87 ^ab^	2.11 ± 0.60 ^abc^	2.00 ± 1.22 ^ab^	2.44 ± 1.01 ^bcd^	1.89 ± 0.93 ^ab^	3.11 ± 1.05 ^d^	3.00 ± 1.12 ^cd^	0.0040 *
Spirtuous	2.22 ± 0.97 ^a^	1.44 ± 1.13 ^ab^	1.22 ± 1.20 ^a^	1.00 ± 0.87 ^a^	1.22 ± 1.09 ^a^	1.33 ± 0.71 ^ab^	0.78 ± 1.09 ^a^	0.67 ± 1.12 ^a^	0.89 ± 0.78 ^a^	1.11 ± 1.45 ^a^	0.1505
Body	1.78 ± 0.67 ^a^	2.89 ± 0.93 ^b^	2.89 ± 1.17 ^b^	2.11 ± 1.17 ^ab^	3.00 ± 1.32 ^b^	2.67 ± 1.00 ^ab^	3.11 ± 0.78 ^b^	2.11 ± 1.54 ^ab^	2.67 ± 1.41 ^ab^	3.00 ± 1.41 ^b^	0.2083

The results represent the mean of all notes given by the tasters for each sensory descriptor and calculated standard deviation. Different superscript letters indicate homogenous groups; * highlights significant differences between samples. P1—Chardonnay, brut nature; P2—Frâncușă, brut; P3—Chardonnay, brut; P4—Grasă de Cotnari, brut; P5—Tămâioasă românească, brut; P6—Chardonnay, dry; P7—Chardonnay, demidry; P8—Grasă de Cotnari, demi dry; P9—Chardonnay, sweet; P10—Tămâioasă românească, sweet.

**Table 3:** Sensory characteristics of rosé sparkling wines.

Descriptors	P11	P12	P13	P14	*p*-Value
Efervescence	3.78 ± 0.97 ^a^	4.00 ± 0.87 ^a^	3.89 ± 0.93 ^a^	4.00 ± 0.71 ^a^	0.9395
Perlage	4.00 ± 0.71 ^a^	4.00 ± 0.71 ^a^	3.78 ± 1.09 ^a^	3.67 ± 1.58 ^a^	0.8866
Plums	1.89 ± 1.36 ^a^	1.00 ± 1.00 ^a^	1.56 ± 1.01 ^a^	2.22 ± 1.64 ^a^	0.2372
Berries	3.11 ± 0.93 ^a^	2.00 ± 1.22 ^a^	3.00 ± 1.32 ^a^	2.67 ± 1.32 ^a^	0.2242
Blueberries	3.00 ± 1.66 ^b^	1.44 ± 1.01 ^a^	1.89 ± 1.36 ^ab^	2.44 ± 0.88 ^ab^	0.0725
Blackberries	3.11 ± 1.45 ^b^	1.44 ± 1.01 ^a^	3.22 ± 1.39 ^b^	3.00 ± 1.58 ^b^	0.0315 *
Fruity	1.11 ± 1.69 ^a^	0.56 ± 0.88 ^a^	1.22 ± 1.72 ^a^	1.00 ± 1.73 ^a^	0.8103
Roses	1.89 ± 1.96 ^a^	1.22 ± 1.48 ^a^	2.56 ± 1.94 ^a^	2.44 ± 1.81 ^a^	0.3944
Spicy	1.56 ± 1.13 ^a^	0.78 ± 1.30 ^a^	1.00 ± 0.87 ^a^	0.78 ± 1.30 ^a^	0.4540
Leaven	1.44 ± 1.13 ^a^	3.00 ± 1.73 ^b^	1.33 ± 1.00 ^a^	1.89 ± 0.60 ^ab^	0.0216 *
Toast	1.44 ± 1.42 ^a^	1.33 ± 1.58 ^a^	1.56 ± 1.13 ^a^	1.78 ± 0.67 ^a^	0.8905
Aroma persistence	3.78 ± 0.83 ^b^	2.22 ± 1.20 ^a^	3.67 ± 1.41 ^b^	3.22 ± 1.39 ^ab^	0.0464 *
Acidity	2.89 ± 1.05 ^a^	2.33 ± 1.41 ^a^	3.00 ± 0.87 ^a^	2.22 ± 1.30 ^a^	0.4114
Sweet	1.22 ± 1.20 ^a^	1.78 ± 0.44 ^a^	3.33 ± 1.73 ^b^	4.00 ± 1.66 ^b^	0.0003 *
Bitter	2.22 ± 1.39 ^b^	2.33 ± 1.58 ^b^	0.67 ± 0.50 ^a^	1.33 ± 1.41 ^ab^	0.0315*
Taste persistence	3.44 ± 1.01 ^a^	2.89 ± 1.05 ^a^	3.11 ± 1.36 ^a^	3.00 ± 1.32 ^a^	0.7818
Astringency	2.89 ± 1.17 ^b^	3.11 ± 1.54 ^b^	1.22 ± 0.97 ^a^	2.44 ± 1.13 ^b^	0.0117 *
Unctuosity	1.56 ± 0.88 ^a^	1.78 ± 0.97 ^a^	2.22 ± 1.30 ^ab^	3.00 ± 1.22 ^b^	0.0462
Spirtuous	1.44 ± 1.01 ^ab^	2.33 ± 1.50 ^b^	1.11 ± 0.93 ^a^	1.11 ± 1.27 ^a^	0.1215
Body	3.33 ± 0.71 ^b^	2.22 ± 0.44 ^a^	2.89 ± 1.05 ^ab^	3.00 ± 1.12 ^ab^	0.0725

The results represent the mean of all notes given by the tasters for each sensory descriptor and calculated standard deviation. Different superscript letters indicate homogenous groups; * highlights significant differences between samples. P11—Fetească neagră, brut; P12—Busuioacă de Bohotin, dry; P13—Fetească neagră, demi dry; P14—Fetească neagră, sweet.

**Table 4:** Characterization of chocolate samples.

Code	Assortment	Ingredients	Nutritional Information (100 g)
C1	White chocolate	Sugar, cocoa butter, whole milk powder, skimmed milk powder, milk fat, lecithin from soy	Fat: 38 g Carbohydrate: 53 g Protein: 6.1 g Salt: 0.2 g
C2	Ruby chocolate	Sugar, cocoa butter, whole milk powder, cocoa mass 40%, sunflower lecithin, citric acid, natural vanilla flavor	Fat: 36.1 g Carbohydrate: 53.1 g Protein: 6.2 g Salt: 0.21 g
C3	Milk chocolate, with 32% cocoa powder	Sugar, cocoa butter, skimmed milk powder, cocoa mass, clarified butter, lecithin from soy, vanilla extract	Fat: 37.9 g Carbohydrate: 50.4 g Protein: 7.3 g Salt: 0.25 g
C4	Dark chocolate, with 70% cocoa powder	Cocoa mass, sugar, cocoa butter, soy lecithin, vanilla extract	Fat: 41.5 g Carbohydrate: 33.8 g Protein: 8.4 g Salt: 0.03 g
C5	Dark chocolate, with 95% cocoa powder	Cocoa mass, cocoa with reduced content of fat, cocoa butter, sugar	Fat: 51.1 g Carbohydrate: 12.7 g Protein: 12.5 g Salt: 0.03 g

**Table 5:** Association degree between sparkling wine and chocolate.

Samples	C1	C2	C3	C4	C5
P1	5.56 ± 1.88 ^ab^	6.56 ± 2.07 ^abc^	5.67 ± 1.73 ^ab^	4.67 ± 2.00 ^ab^	3.11 ± 2.26 ^abc^
P2	6.50 ± 1.31 ^ab^	7.25 ± 2.05 ^abc^	7.38 ± 1.19 ^cd^	3.50 ± 1.20 ^a^	2.13 ± 0.99 ^a^
P3	6.89 ± 2.03 ^ab^	6.44 ± 1.81 ^ab^	6.78 ± 1.86 ^bc^	3.56 ± 1.59 ^a^	2.56 ± 1.67 ^ab^
P4	7.11 ± 1.62 ^b^	5.67 ± 2.12 ^a^	5.44 ± 1.13 ^ab^	6.56 ± 0.53 ^c^	2.33 ± 1.58 ^a^
P5	7.00 ± 1.66 ^ab^	6.67 ± 1.80 ^abc^	6.11 ± 1.62 ^abc^	4.33 ± 2.00 ^a^	2.33 ± 1.50 ^a^
P6	6.22 ± 1.72 ^ab^	7.11 ± 1.05 ^abc^	6.22 ± 1.72 ^abc^	4.44 ± 1.94 ^a^	3.22 ± 1.86 ^abcd^
P7	7.22 ± 0.83 ^b^	7.56 ± 1.51 ^bc^	6.44 ± 1.51 ^abc^	6.22 ± 2.17 ^bc^	4.33 ± 2.50 ^bcde^
P8	6.22 ± 2.11 ^ab^	7.33 ± 1.94 ^abc^	6.78 ± 1.48 ^bc^	6.44 ± 2.46 ^c^	5.00 ± 2.56 ^de^
P9	6.67 ± 2.29 ^ab^	6.00 ± 1.73 ^ab^	6.22 ± 1.99 ^abc^	6.78 ± 2.91 ^cd^	5.44 ± 2.74 ^ef^
P10	6.89 ± 1.83 ^ab^	6.78 ± 1.79 ^abc^	8.73 ± 1.12 ^d^	6.56 ± 2.46 ^c^	5.11 ± 2.80 ^ef^
P11	5.33 ± 2.24 ^a^	6.33 ± 2.55 ^ab^	6.33 ± 2.18 ^abc^	7.89 ± 1.22 ^cd^	6.89 ± 1.01 ^fg^
P12	5.89 ± 1.27 ^ab^	6.89 ± 1.36 ^abc^	5.11 ± 1.45 ^a^	7.56 ± 1.13 ^cd^	4.89 ± 1.05 ^cde^
P13	6.11 ± 2.42 ^ab^	8.22 ± 1.30 ^c^	7.44 ± 1.74 ^cd^	7.00 ± 1.12 ^cd^	6.00 ± 2.00 ^ef^
P14	6.11 ± 1.62 ^ab^	7.11 ± 1.62 ^abc^	6.67 ± 1.50 ^bc^	8.33 ± 0.71 ^d^	8.11 ± 0.93 ^g^
*p*-value	0.5382	0.3104	0.0063 *	0.0000 *	0.0000 *

Different letters indicate significant differences according to post hoc comparison at a significance level of *p* ≤ 0.05, while * highlights a significant difference of perception. C1—white chocolate; C2—ruby chocolate; C3—milk chocolate, with 32% cocoa powder; C4—dark chocolate, with 70% cocoa powder; C5—dark chocolate, with 95% cocoa powder.

**Table 6:** Coefficient of determination—summary for all Ys.

Title 1	C1	C2	C3	C4	C5	C1
R^2^	0.096969	0.120563	0.221552	0.44393	0.490495	R^2^
F	0.916873	1.170543	2.43011	6.816559	8.21988	F
Pr > F	0.538	0.310	0.006	<0.0001	<0.0001	Pr > F

C1—white chocolate; C2—ruby chocolate; C3—black chocolate, with 32% cocoa powder; C4—black chocolate, with 70% cocoa powder; C5—black chocolate, with 95% cocoa powder.

**Table 7:** Wilks’ Lambda test (Rao’s approximation).

Parameters	Values
Lambda	0.228
F (Observed value)	2.883
F (Critical value)	1.332
DF1	65
DF2	509.606
*p*-value (Two-tailed)	<0.0001
alpha	0.05

## Data Availability

The data used to support the findings of this study can be made available by the corresponding author upon request.
